# The Safety and Treatment Response of Combination Therapy of Radioimmunotherapy and Radiofrequency Ablation for Solid Tumor: A Study *In Vivo*

**DOI:** 10.1371/journal.pone.0096539

**Published:** 2014-05-02

**Authors:** Shu-Guang Zheng, Hui-Xiong Xu, Le-Hang Guo, Lin-Na Liu, Feng Lu

**Affiliations:** 1 Department of Medical Ultrasound, Shanghai Tenth People’s Hospital, Tenth People’s Hospital of Tongji University, Shanghai, China; 2 Department of Medical Ultrasonics, The First Affiliated Hospital, Sun Yat-Sen University, Guangzhou, China; Johns Hopkins University, United States of America

## Abstract

**Objection:**

To investigate the safety and treatment response of radioimmunotherapy (RIT) in combination with radiofrequency ablation (RFA) for the treatment of VX_2_ tumor on rabbit.

**Materials and Methods:**

A total of 36 rabbits bearing VX_2_ tumor on the thigh were randomly assigned into 3 groups (group I: 1–2 cm; group II: 2–3 cm; group III: 3–4 cm) and 4 subgroups (A: as control, just puncture the tumor using the RFA electrode without power output; B: RFA alone; C: ^131^I-chTNT intratumoral injection alone; D: RFA+^131^I-chTNT intratumoral injection 3 days later). The variation of blood assay, weight and survival among different groups and subgroups were used to assess the treatment safety. Ultrasound (US) was used to monitor and assess the tumor response after treatment.

**Results:**

According to the results of the weight and the blood assay among different groups, subgroups, and at two time points (one day before and the 16th day after treatment), no damages to the liver, kidney function and myelosuppression resulting from the treatment were found. No significant differences in survivals among the four subgroups (*p* = 0.087) were found. In addition, ^131^I-chTNT did not show significant inhibition effect on VX_2_ tumor progression according to US measurements.

**Conclusion:**

^131^I-chTNT intratumoral injection alone or in combination with RFA is relatively safe for rabbit without significant toxicity and shows no significant effect on the survival. The treatment response is not as satisfactory as anticipated.

## Introduction

Radioimmunotherapy (RIT) is a powerful and attractive tool for the treatment of local and/or diffuse tumors with radiation [1], [2]. The use of antibody conjugated radionuclides firstly started in the early 1950s [3]. Recently, the major advance in RIT is the treatment of hematological malignances that the FDA has approved use of ^90^Y ibritumomab tiuxetan (Zevalin) and ^131^I tositumomab (Bexxar) in treating non-Hodgkins’ lymphoma (NHL) [4]–[7].

However, for solid tumors, RIT is still challenging and only few clinical trials have gone beyond Phase II, which stimulates more researchers to explore this issue [1], [8]. Song *et al* [4] proposed that two therapeutic components: radiation delivery and antibody action upon binding were the main contribution to kill cancer cells and to enhance the overall tumor response; Huang *et al* [9] also mentioned that therapeutic efficacy of RIT to solid tumor was limited by the inadequate delivery and penetration of RIT agents into solid tumor. Thus, most of recent studies have focused on how to improve the tumor uptake [10]. Whether the higher targeting concentration of RIT agents will acquire better response for solid tumors is still controversial.

In a previous study on rabbit bearing VX_2_ tumor, RFA before intratumoral injection of ^131^I-chTNT dramatically improving the targeting concentration (the tumor to normal tissue ratio [T/NT] from 7.63±5.61 to 55.45±41.83) had been demonstrated [11]. The VX_2_ tumor is an anaplastic squamous cell carcinoma derived from a virus-induced papilloma in the wild rabbit, but appears as a carcinoma in the domestic species. Tumor necrosis therapy (TNT) uses degenerating tumor cells and necrotic regions of tumors as targets for RIT. ^131^I-chTNT is an 131-iodine radiolabeled chimeric monoclonal antibody (MAb) targeted against intracellular DNA exposed in necrotic and degenerating regions of malignant solid tumors, which has shown some promising applications in some clinical trials for treatment of lung cancer, glioblastoma, colorectal carcinoma, and other malignant solid tumors [8], [11]–[13]. Radiofrequency ablation (RFA) is also a validated therapeutic modality for the treatment of some solid tumors of the whole body [14]–[17]. Then as far as intratumoral injection of ^131^I-chTNT in combination with RFA was concerned, whether therapeutic efficacy could be improved along with the improvement of RIT agent targeting concentration and how about their safety are worth to be further investigated. On the basis of the above-mentioned, this study was aimed to assess the treatment efficacy and safety of intratumoral injection of ^131^I-chTNT in combination with RFA for the treatment of VX_2_ tumor on rabbit.

## Materials and Methods

### Preparation of the Animal Model

All animal experiments were approved by animal research ethic committee of Sun Yat-sen University, China. During all the procedures, animal welfare was carried out according to the ARRIVE (Animal Research: Reporting of In Vivo Experiments) Guidelines. A total of 36 New Zealand white rabbits weighting 2.33±0.32 kg (range, 1.9–2.9 kg) and bearing VX_2_ tumor on the thigh were enrolled in this study. The rabbits were purchased from the laboratory animal center of Guangdong and the VX_2_ tumor was obtained from Laboratory Animal Center, Sun Yat-sen University, China.

All the rabbits were randomly and equally assigned into 3 groups (12 rabbits in each group). When the VX_2_ tumor grew up to the anticipated size (group I: 1–2 cm, group II: 2–3 cm, group III: 3–4 cm), each group was further divided into 4 subgroups (3 rabbits in each subgroup) randomly to receive treatments as follows:

Subgroup A: as control, just puncture the tumor using the RFA electrode without power output;
Subgroup B: RFA alone;
Subgroup C: 131I-chTNT intratumoral injection alone;
Subgroup D: RFA+131I-chTNT intratumoral injection 3 days later.

### Treatments

All rabbits received a solution of potassium iodine orally, beginning from 3 days before treatment and lasting to the 15th day after treatment, to block uptake of free iodine-131 by their thyroid. Before treatment, the rabbits were anesthetized by injection with 3% pentobarbital solution (1 mL/kg) via ear vein and were fixed on the bed in left lateral decubitus; the thighs of the rabbits were shaved and prepared with povidone iodine. US machine (M-Turbo Ultrasound system, Sonosite Inc, Bothell, Washington, USA) equipped with a transducer with frequency of 13-6 MHz was performed in the experiments for puncture guidance. All treatments were performed under sterile condition.

### RFA

RFA procedures were performed using a Cool-tip system (Valleylab, Boulder, Colo, USA), which consists of an RF generator with a maximum power of 200 W, a 20 cm-long 17-gauge internally cooled electrode with a 3 cm active tip, and a dispersive ground pad was attached on the shaved back of the rabbits. Under the guidance of US, the RFA electrode was inserted into the VX_2_ tumor along the longest diameter with energy output of 50 w for 3 min.

### ^131^I-chTNT

^131^I-chTNT (Vivatuxin, Shanghai Medipharm Biotech Co. Ltd, Shanghai, China) is a radiolabeled recombinant human-mouse chimeric TNT (ch-TNT) antibody. The purified ch-TNT antibody with purity of at least 98% is radiolabeled with Na^131^I, which has a radioactive range of 2.2 mm in the tissue and a half-life of 8 days. The purity of ^131^I-chTNT is over 95% with a specific radioactivity of 10 mCi/mL (370 MBq/mL).

^131^I-chTNT was injected into the VX_2_ tumor by using a 22-gauge fine core needle with a recommended dose of 1.4 mCi/cm^3^ VX_2_ tumor from the manufacturer. This dose was calculated according to the reported formula for dose translation based on the body surface area by Reagan-Shaw et al [18]. The US guidance made sure of the accurate placement of the needle at multiple sites (0, 3, 6, 9 o’clock direction) and simultaneously monitored the ^131^I-chTNT injection and observed its distribution in the VX_2_ tumor. After injection of ^131^I-chTNT, 0.5 mL normal saline was used to flush the needle. The puncture site was then gently compressed using alcoholic cotton gauze for 2 min to avoid leakage of ^131^I-chTNT or bleeding after removal of the needle.

### SPECT/CT Fusion Imaging

At the first day after ^131^I-chTNT injection, the rabbits (in the subgroup C and D) underwent SPECT/CT scanning for acquiring fusion image to observe the distribution of ^131^I-chTNT. The acquisition parameters of SPECT/CT (Symbia T2 SPECT/CT system, Siemens Munich, Germany) were used as follows: zoom 1.5, a 128×128 matrix with a pixel acquisition, the fusion CT acquisition used full-circle rotation, 130 kV, 35 mAs, and 5-mm slices.

### Safety and Survival

The treatment safety for the rabbit was assessed mainly on the basis of blood assay, weight and survival. At two time points (1 day before and the 16th day after treatment), the blood samples were drawn from the ear artery of the rabbits for testing the count of blood cell (CBC) (including: white blood cell, WBC; red blood cell, RBC; hemoglobin, HB; platelet, PLT), liver function (including: alkaline phosphates, ALP; aspartate transaminase, AST; alanine aminotransferase, ALT; total protein, TP; albumin, ALB;) and kidney function (including: blood urea nitrogen, BUN; creatinine, Cr). Simultaneously, all rabbits were weighed. After treatment, all rabbits were raised and checked twice every day for observing the survival. The humane endpoint was defined by >50% decrease in food intake and >20% loss in body weight. Then they were humanely euthanized by injection of overdose 3% pentobarbital solution (>3 mL/kg) via ear vein.

### Treatment Response and Progression after Treatment

During the follow-up period after treatment, US was performed by two operators independently for measuring the three greatest dimensions (x, y, z) at 3 days interval until the 15th day. Meanwhile the tumor volumes (TV) were calculated according to the following formula:π•x•y•z/6. The mean TV was used for evaluating the treatment response.

On the basis of the TV, The tumor volume growth rate (TVGR) at n day and the tumor volume growth rate at each interval (TVGRI) were calculated based on the following equations:
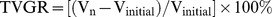


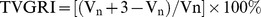
V_n_ and V_initial_ were the tumor volume of each group measured at n day and start of the treatment respectively.

Finally, on the basis of the TV, the tumor growth curve was drawn. The treatment response and tumor progression were evaluated by comparing the TV, TVGR and TVGRI.

### Statistical Analysis

Continuous data were expressed as mean ± standard deviation. Multiple comparisons were performed using univariate/repeated measures analysis of variance (ANOVA) with Bonferroni-test. Kaplan-Meier survival curves for overall survival were generated, and the log-rank test was used to identify the difference between the subgroups. The level of statistical significance was set at *P*<0.05. SPSS software (version 16.0, SPSS Inc., Chicago, IL, USA) was used to perform the statistical analysis.

## Results

**Figure 1:**
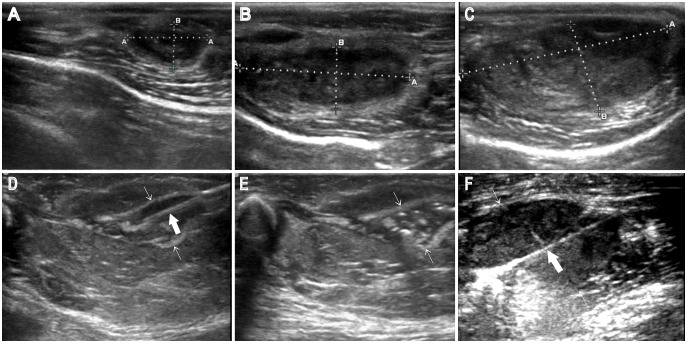
The ultrasound images of VX_2_ tumor and puncturing guidance. A, B, C: a VX_2_ tumor on ultrasound (US) at 0, 6th and 12th day after treatment, respectively; D: ^131^I-chTNT intratumoral injection; E: after ^131^I-chTNT intratumoral injection; F: radiofrequency ablation (RFA). Thin arrow: the VX_2_ tumor; thick arrow: PTC needle (D) or RFA electrode (F).

During the whole experimental procedure, US was used to measure the VX_2_ tumor size (Figure 1. A, B, C), guide percutaneous RFA electrode insertion and monitor the treatment procedure (Figure 1. D, E, F).

**Figure 2:**
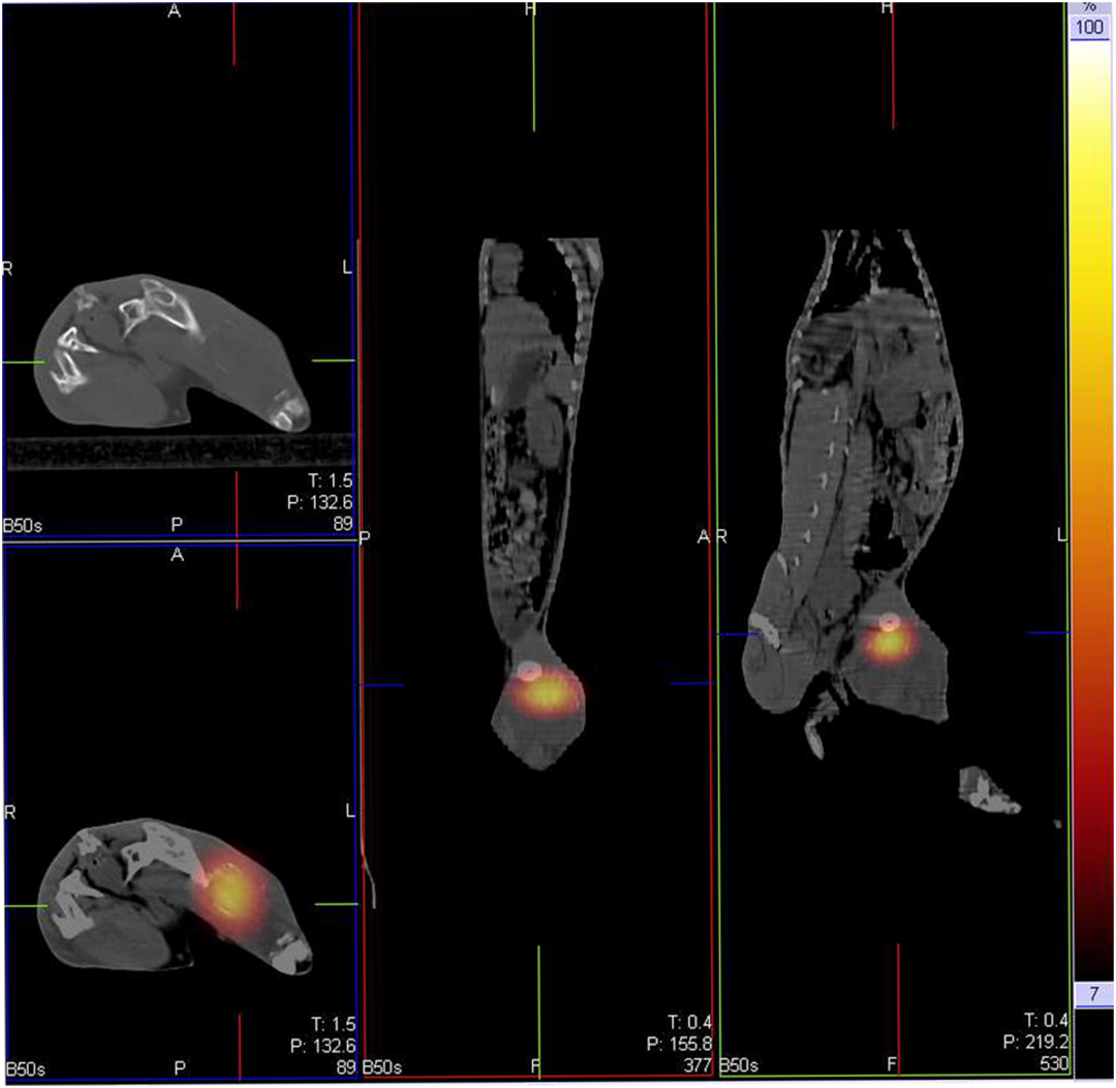
The SPECT/CT fusion images. The fusion images show the injected ^131^I-chTNT overlaps with the tumor.

Until the 16th day after the treatment, no severe complications or death were encountered. On SPECT/CT fusion image at the first day after intratumoral injection,^ 131^I-chTNT was observed overlapping the VX_2_ tumor with a high concentration (Figure 2).

The results of the weight and the blood assay including CBC, liver and kidney function at two time-points (one day before and the 16th day after treatment) were listed in Table 1. Based on the analysis results of the above-mentioned parameters, no significant differences were found among different groups (all *p*>0.05); No significant differences were found among different subgroups (all *p*>0.05), except for BUN, Cr, ALP and ALB (all *p*<0.05); Additionally, no significant differences were found between the two time-points (all *p*>0.05), except for ALB, WBC and PLT(all *p*<0.05). Thus no damages to the liver, kidney function and myelosuppression resulting from the treatment were found.

**Table 1:** The results of the weight and the blood assay of rabbit before and after treatment.

Parameters	Timepoints	I				II			III				
		A	B	C	D	A	B	C	D	A	B	C	D
Weight (kg)	before	2.03±0.06	2.67±0.12	2.37±0.25	2.60±0.26	2.00±0.10	2.83±0.12	2.07±0.12	2.50±0.20	2.13±0.21	2.47±0.15	2.00±0.10	2.40±0.20
	after	2.20±0.10	2.63±0.06	2.70±0.26	2.63±0.25	2.23±0.06	2.97±0.15	2.27±0.25	2.57±0.15	2.30±0.17	2.57±0.31	2.07±0.06	2.53±0.15
BUN(mmol/L)	before	8.17±0.67	8.67±5.52	6.57±0.84	4.67±1.00	8.53±0.91	6.77±1.31	6.50±2.31	3.07±0.70	8.63±0.56	8.00±2.40	6.57±0.95	3.93±0.70
	after	9.03±1.01	7.90±3.25	6.40±2.62	8.90±1.80	8.83±0.70	6.97±0.67	4.70±1.97	6.80±1.42	9.70±1.05	9.80±1.05	4.10±0.36	8.93±2.40
Cr(umol/L)	before	61.33±12.66	70.00±20.95	63.67±9.87	54.67±5.86	71.00±6.08	68.67±5.03	60.67±20.50	37.67±2.89	67.00±3.00	61.33±4.93	63.00±10.44	55.33±8.62
	after	64.67±10.97	67.33±16.44	55.33±5.86	66.33±6.66	74.00±2.00	73.67±11.06	53.00±4.36	39.33±9.61	75.33±7.02	74.00±15.88	49.33±3.79	42.67±11.85
ALP (U/L)	before	150.67±37.67	101.33±72.71	111.33±38.01	90.67±2.08	149.67±14.36	112.67±43.50	100.00±59.09	98.67±8.33	132.33±10.69	57.00±50.39	94.00±22.07	102.00±36.37
	after	99.33±22.01	56.33±25.01	49.00±24.25	74.33±37.53	85.00±22.52	69.67±19.43	69.00±31.43	34.00±12.12	58.33±21.03	38.00±1.00	43.00±21.93	34.33±2.89
AST (U/L)	before	26.60±3.29	18.00±3.00	17.27±3.19	35.97±17.76	28.00±6.88	39.07±17.86	16.03±6.73	80.57±58.35	23.63±7.28	20.50±5.70	20.43±5.85	50.73±25.18
	after	41.57±15.56	20.47±4.41	30.57±15.44	59.90±69.97	32.53±2.59	32.83±5.11	21.90±6.05	20.97±5.97	44.87±10.43	45.13±9.76	13.40±4.43	25.73±12.80
ALT (U/L)	before	34.03±6.01	34.00±7.55	47.67±8.01	41.30±13.72	42.10±17.96	48.57±25.07	48.90±23.32	76.53±18.87	27.23±6.66	43.73±15.95	36.93±5.65	47.93±15.03
	after	31.87±14.02	33.00±7.88	35.20±4.32	76.87±65.76	28.07±13.76	44.43±18.67	26.43±2.70	36.33±8.81	28.90±9.73	29.77±12.92	31.30±10.73	30.93±7.01
TP (g/L)	before	58.73±3.33	56.90±3.05	57.00±2.52	55.83±7.35	55.47±1.79	52.27±6.91	55.20±6.04	51.00±0.82	57.30±1.39	44.80±1.73	52.27±3.23	51.67±3.21
	after	59.40±1.85	54.33±2.89	54.67±0.15	60.07±4.13	56.90±4.15	58.33±1.14	56.00±3.51	55.83±3.97	58.90±4.30	60.37±1.56	55.70±1.74	58.47±1.92
ALB(g/L)	before	38.20±1.59	35.80±2.62	37.27±2.30	33.60±1.99	37.00±0.40	33.50±4.76	34.20±0.53	34.70±0.79	38.87±0.40	30.30±2.27	31.70±0.40	33.37±0.80
	after	39.13±0.38	35.90±5.85	34.37±2.06	34.53±2.49	38.37±1.67	37.87±1.93	33.87±3.40	34.23±2.68	36.87±3.91	36.57±1.05	32.63±3.55	32.37±3.16
WBC(10^9^/L)	before	9.80±0.28	9.41±4.52	7.78±2.19	9.35±2.32	7.37±0.90	7.94±1.46	10.18±5.03	10.94±2.06	9.28±3.68	7.30±2.53	8.06±2.47	10.99±1.79
	after	12.39±3.50	6.84±1.46	14.61±6.45	12.64±3.98	15.71±5.69	9.95±0.40	10.57±3.32	12.27±4.38	19.88±8.13	18.41±6.78	13.10±7.45	17.21±4.17
RBC(10^12^/L)	before	5.34±0.25	5.26±1.34	5.50±0.47	4.82±0.16	6.12±0.42	7.08±1.66	5.10±1.04	5.53±0.62	5.58±0.55	6.48±0.57	5.18±0.31	5.11±0.20
	after	5.92±0.06	5.49±1.12	5.44±0.17	5.37±1.03	6.22±0.32	5.82±0.55	5.28±0.60	5.71±0.87	5.83±0.10	5.24±0.44	5.17±1.20	4.54±0.95
PLT(10^9^/L)	before	459.33±232.97	381.33±82.57	482.67±124.68	516.33±61.33	342.67±205.52	318.00±170.00	366.00±340.55	499.00±179.14	466.33±1.53	439.67±88.08	340.67±103.73	383.67±82.58
	after	474.67±147.72	500.33±69.17	899.33±122.17	873.67±388.72	612.00±198.44	521.67±51.07	559.67±222.37	479.00±331.88	700.33±83.76	547.33±158.09	507.67±329.18	615.00±196.54
HB (10^9^/L)	before	115.33±7.37	113.67±16.28	123.00±10.54	107.67±2.51	126.00±6.25	122.33±10.26	105.33±24.09	120.67±9.86	134.67±27.32	113.00±9.00	111.00±4.58	115.33±5.51
	after	124.00±1.73	116.67±28.04	111.67±3.06	103.00±27.22	125.00±3.00	121.00±10.15	110.67±3.78	115.33±17.93	123.33±7.02	112.67±9.87	100.67±26.50	77.67±28.29

I, II and III represent the No. of groups; A, B, C and D represent the No. of subgroups;

Time point: before and after treatment at two time points (1 day before and the 16th day after treatment).

Alkaline Phosphates, ALP; Aspartate Transaminase, AST; Alanine Aminotransferase, ALT; Total Protein, TP; Albumin, ALB; Blood Urea Nitrogen, BUN; Creatinine, Cr; White Blood Cell, WBC; Red Blood Cell, RBC; Hemoglobin, HB; Platelet, PLT.

**Figure 3:**
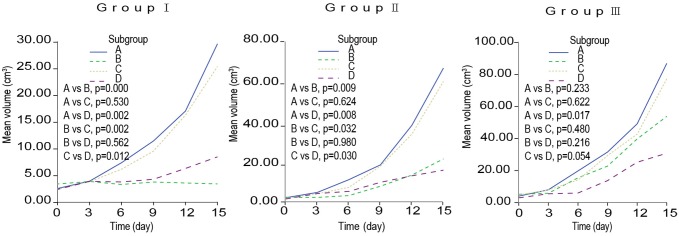
The growth curves of the tumors after treatment in group I, II, III. Multiple comparisons among different subgroups (Bonferroni-test).

According to US measurement results during the follow-up from the treatment beginning to the 15th day after treatment, the TV, TVGR and TVGRI were calculated and listed in Table 2 and Table 3, respectively. According to the analysis results, all data were normally distributed and met the homogeneity of variance. Accordingly, the growth curve of the tumor in each group was drawn (Figure 3). Regarding TVGR and TVGRI, no significant differences were found among different groups (all *p*>0.05); but both parameters between subgroup A and B, A and D, B and C, and C and D, showed significant difference (all *p*<0.05). Obviously, unlike RFA, ^131^I-chTNT had no significant inhibition effect on VX_2_ tumor progression.

**Table 2:** The tumor volumes (TVs) of VX_2_ tumors during the follow-up.

Group	Subgroup	TV (cm^3^)					
		0 day	3 day	6 day	9 day	12 day	15 day
I	A	0.88±0.42	2.24±0.60	5.70±2.70	9.78±2.34	15.38±0.68	27.97±2.63
	B	1.71±0.86	2.19±1.42	1.63±0.89	2.08±2.02	1.90±1.41	1.73±1.62
	C	0.71±0.18	2.14±0.84	4.48±2.08	7.87±3.31	14.61±3.44	23.71±6.80
	D	0.70±0.32	2.20±1.13	2.09±1.22	2.60±1.62	4.62±2.25	6.80±2.80
II	A	1.96±0.48	4.39±1.97	10.23±2.25	17.00±2.92	35.16±5.66	61.35±5.50
	B	2.13±0.51	2.09±0.51	2.92±0.53	7.22±4.98	12.33±8.27	19.74±15.44
	C	1.55±0.30	3.79±1.04	6.71±0.33	16.37±5.16	31.13±5.43	55.19±4.85
	D	1.71±0.22	3.86±1.00	4.85±1.81	8.99±1.49	11.99±4.33	14.58±4.81
III	A	4.01±1.10	7.98±1.07	19.75±4.05	31.58±7.13	49.20±6.31	86.98±16.53
	B	5.18±1.99	5.83±0.54	15.42±7.80	22.79±8.17	39.77±12.80	53.86±10.67
	C	4.77±2.72	7.84±1.66	14.12±4.51	29.39±13.52	42.76±17.55	77.35±22.07
	D	3.95±0.82	5.49±2.00	5.96±1.78	13.72±6.69	25.21±9.58	30.83±9.74

**Table 3:** The tumor volume growth rate (TVGR) and the tumor volume growth rate at each interval (TVGRI) of VX_2_ tumors during the follow-up.

Group	Subgroup	TVGR,% (TVGRI,%)				
		3 day	6 day	9 day	12 day	15 day
I	A	1.76±0.65	5.85±2.34	11.62±5.70	19.89±11.01	36.29±18.44
		(1.76±0.65)	(1.49±0.68)	(0.83±0.38)	(0.63±0.36)	(0.82±0.17)
	B	0.66±1.34	0.18±0.90	0.57±1.76	0.39±1.30	0.29±1.42
		(0.66±1.34)	(−0.17±0.29)	(0.11±0.48)	(0.06±0.23)	(−0.17±0.16)
	C	1.92±0.46	5.09±1.38	9.74±1.96	19.52±1.52	32.43±7.41
		(1.92±0.46)	(1.08±0.28)	(0.77±0.11)	(0.96±0.40)	(0.62±0.23)
	D	1.09±0.83	1.84±0.59	2.63±0.81	5.55±1.56	9.37±3.45
		(1.09±0.83)	(−0.09±0.19)	(0.32±0.39)	(0.93±0.91)	(0.59±0.44)
II	A	1.17±0.49	4.25±1.09	8.08±2.99	18.03±7.39	31.26±5.92
		(1.17±0.49)	(1.50±0.59)	(0.73±0.54)	(1.08±0.15)	(0.78±0.36)
	B	0.06±0.51	0.41±0.35	2.33±1.86	4.632.84	7.81±5.13
		(0.06±0.51)	(0.49±0.62)	(1.32±0.26)	(0.78±0.28)	(0.54±0.19)
	C	1.46±0.56	3.44±0.83	9.81±3.54	19.42±3.92	35.44±7.19
		(1.46±0.56)	(0.84±0.36)	(1.42±0.65)	(0.97±0.36)	(0.82±0.41)
	D	1.24±0.41	1.91±1.26	4.35±1.33	6.21±3.31	7.61±3.28
		(1.24±0.41)	(0.33±0.56)	(0.96±0.42)	(0.36±0.52)	(0.24±0.31)
III	A	1.04±0.28	4.34±2.25	7.38±3.02	11.83±3.27	21.26±4.31
		(1.04±0.28)	(1.54±0.78)	(0.61±0.22)	(0.59±0.23)	(0.76±0.20)
	B	0.27±0.58	1.85±0.61	3.48±0.59	6.83±1.01	10.11±2.91
		(0.27±0.58)	(1.68±1.46)	(0.66±0.61)	(0.78±0.39)	(0.41±0.25)
	C	0.95±1.02	2.97±3.12	7.65±7.67	11.38±10.59	20.66±16.21
		(0.95±1.02)	(0.85±0.61)	(1.03±0.33)	(0.48±0.11)	(0.86±0.21)
	D	0.45±0.66	0.57±0.62	2.78±2.24	5.86±3.51	7.33±3.82
		(0.45±0.66)	(0.10±0.06)	(1.34±1.26)	(0.96±0.37)	(0.25±0.11)

**Figure 4:**
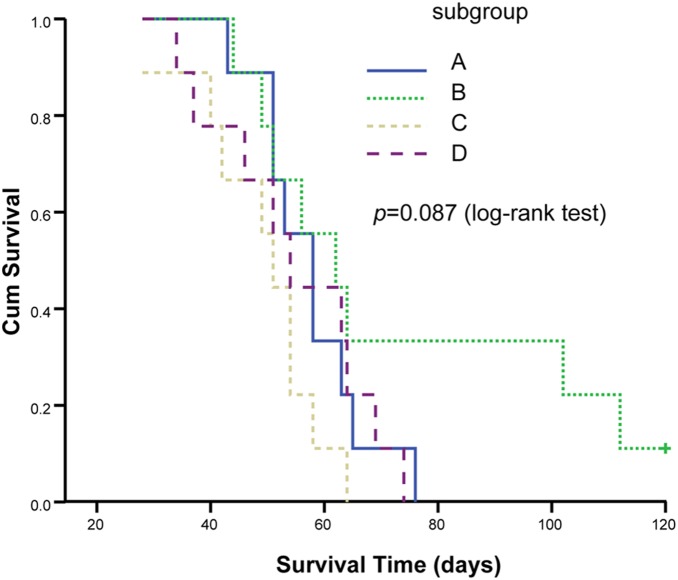
The survival curves of the rabbits in four subgroups. Multiple comparisons among different subgroups (p = 0.087, log-rank test).

After treatment, all rabbits were followed up until the 120th day and finally were humanely euthanized when they met the above-mentioned criteria of the endpoint, except for 2 rabbits in subgroup B of group I. Subsequently, the survival curves were drawn on the basis of the survivals of the rabbits (Figure 4). The log-rank test revealed that there was no significant difference among the four subgroups (*p* = 0.087).

## Discussion

Enhancing the overall response/survival and decreasing the toxicity are always the main pursues of RIT for treatment of various solid tumors. In the past decades, much effort has been focused on the development of the best targeting vector, choosing the most appropriate isotope, and finding the suitable administration procedure [1], [2], [4], [10], [19]–[22].

It is reported that multiple barriers to delivery still exist which prevent effective immunotherapy for solid tumors in vivo, such as hemodilution, renal excretion, neutralization of free antibodies and block of vascular endothelial cell, physical decay, short radiation range and duration, and so on [1], [2], [4], [9], [19]. Hence a series of antibodies with higher active targeting ability have been explored for the application of RIT; meanwhile many strategies including the pre-targeting technique, administration via tumorous supply artery, and intratumoral injection, have been employed [1], [2], [9], [21]–[24].

Recently, it is proposed that RIT could be combined with other treatments to enhance the efficacy [1], [2], [24]–[28]. For instance, US-guided percutaneous local treatments are playing increasingly important roles for a variety of solid tumors. Barbet *et.al* [20] mentioned that the loco-regional route is preferable for the radionuclides when targeted by antibodies because using the intravenous route always results in significant radionuclide decay before the antibody has reached its target. Therefore, US guided ^131^I-chTNT intratumoral injection in combination with RFA was performed in this study.

Theoretically, a higher targeting concentration of RIT agents, will achieve a better treatment response and a less toxicity. Chen *et al* [8] reported a phase II clinical trial conducted in China on 107 patients with advanced lung cancer. ^131^I-chTNT (0.8mCi/kg) was administered by intravenous injection at 2–4 weeks interval (n = 62) and intratumoral injection (n = 45). At 10 weeks post-treatment, in spite of some mild side effects and reversible bone marrow suppression, the intratumoral injection (less hematologic toxicity) was proven to be safer than intravenous injection. The overall response rates were up to 35.5%. Unfortunately, no evidence of benefit to the overall survival was documented [8].

In this study, US guided direct ^131^I-chTNT intratumoral injection was used to avoid the hemodilution and RFA was performed in advance to increase the targeting site of ^131^I-chTNT. The fused SPECT/CT image on the first day after treatment also proved a high targeting concentration of 131-iodine. No significant differences regarding the hematologic toxicity of ^131^I-chTNT, as well as weight, liver and kidney function, were found among different subgroups. Furthermore, the post-treatment survival among different subgroups also did not show significant difference. That is to say, ^131^I-chTNT with or without RFA was safe enough for rabbit.

However, the treatment response of VX_2_ tumor was not as expected as that of the lung cancer [8]. As mentioned above, RFA is a validated therapeutic modality for the treatment of some solid tumors. On the other hand, ^131^I-chTNT was not found effective for the treatment of VX_2_ tumor, whether used alone (all Subgroup A vs. C, *p*>0.05) or combined with RFA (all Subgroup B vs. D, *p*>0.05), despite that RFA in advance can dramatically improve the targeting concentration of ^131^I-chTNT after intratumoral injection [11]. Thus improving the targeting concentration of RIT agents alone seems inadequate for RIT of some kinds of solid tumors. In the case of RIT, as Navarro-Teulon *et al* [1] have mentioned, solid tumors are more radio-resistant than hematological malignancies. Hannaoka *et al* [29] also concluded that the therapeutic efficacy of RIT seems largely dependent on the tumor radiosensitivity. Therefore the radiosensitivity should be regarded as the fundamental element responsible for treatment response of solid tumors, besides the targeting MAbs, isotopes and administration modalities. Thus, further studies should be focused on how to enhance the tumor response.

There were some limitations in this study as follows: firstly, just VX_2_ tumor model on rabbits was used without other tumor model as comparison. Furthermore, no various dose of ^131^I-chTNT were performed to investigate the VX_2_ tumor response and the sensitive dose. Additionally, although no significant differences in survival were found, the Kaplan-Meier survival analysis showed that the subgroup that received RFA only differed quite substantially from the others, possibly due to the hematological toxicity in the RIT subgroups. Finally, the blood assay was just performed at two time points without further comparison at longer time point.

## Conclusion

In summary, ^131^I-chTNT intratumoral injection alone or in combination with RFA is relatively safe for rabbit without significant toxicity and shows no significant effect on the survival. In addition, the treatment response for the combined therapy was not as satisfactory as anticipated.
